# Maslinic Acid, a Triterpene from Olive, Affects the Antioxidant and Mitochondrial Status of B16F10 Melanoma Cells Grown under Stressful Conditions

**DOI:** 10.1155/2015/272457

**Published:** 2015-07-07

**Authors:** Khalida Mokhtari, Eva E. Rufino-Palomares, Amalia Pérez-Jiménez, Fernando J. Reyes-Zurita, Celeny Figuera, Leticia García-Salguero, Pedro P. Medina, Juan Peragón, José A. Lupiáñez

**Affiliations:** ^1^Department of Biochemistry and Molecular Biology I, Faculty of Sciences, University of Granada, 18071 Granada, Spain; ^2^Department of Microbiology, Faculty of Sciences, Mohammed I University of Oujda, 60000 Oujda, Morocco; ^3^Department of i+D+I, Biomaslinic S.L., Polígono Industrial de Escúzar, 18130 Granada, Spain; ^4^Department of Biochemistry, Faculty of Sciences, Simón Bolívar University of Caracas, Caracas 1080, Venezuela; ^5^Department of Experimental Biology, Biochemistry Section, Faculty of Experimental Biology, University of Jaén, 23071 Jaén, Spain

## Abstract

Maslinic acid (MA) is a natural compound whose structure corresponds to a pentacyclic triterpene. It is abundant in the cuticular lipid layer of olives. MA has many biological and therapeutic properties related to health, including antitumor, anti-inflammatory, antimicrobial, antiparasitic, antihypertensive, and antioxidant activities. However, no studies have been performed to understand the molecular mechanism induced by this compound in melanoma cancer. The objective of this study was to examine the effect of MA in melanoma (B16F10) cells grown in the presence or absence of fetal bovine serum (FBS). We performed cell proliferation measurements, and the reactive oxygen species (ROS) measurements using dihydrorhodamine 123 (DHR 123) and activities of catalase, glucose 6-phosphate dehydrogenase, glutathione S-transferase, and superoxide dismutase. These changes were corroborated by expression assays. FBS absence reduced cell viability decreasing IC_50_ values of MA. The DHR 123 data showed an increase in the ROS level in the absence of FBS. Furthermore, MA had an antioxidant effect at lower assayed levels measured as DHR and antioxidant defense. However, at higher dosages MA induced cellular damage by apoptosis as seen in the results obtained.

## 1. Introduction

*Olea europaea* L. is an evergreen tree widely distributed in the Mediterranean countries, where they cover 8 million ha, accounting for almost 98% of the world crop [1]. It is studied for its alimentary use (the fruits and the oil are important components in the daily diet of a large part of the world's population), whereas the leaves and seeds are important for their secondary metabolites such as terpenes group.

Maslinic acid (MA), a natural pentacyclic triterpene, has attracted much interest due to its proven pharmacologic safety and its many biological activities, such as antiviral [2], antidiabetogenic [3], anti-inflammatory [4], and antimicrobial [5] functions. More recently, some studies have shown that MA has anticancer property in different types of cancer [6–12]. Moreover, MA inhibits glycogen phosphorylase in rat liver and muscle [13–15], decreases glucose in diabetogenic mouse [16], and stimulates healthy whole animal and tissue growth [17–21]. Although a recent study showed that MA induces apoptosis in several cancer cells no such effect has been reported in melanoma cells. In particular, the involvement of MA-mediated reactive oxygen species (ROS) production in apoptotic signaling in B16F10 melanoma cells remains unknown. Oxidative stress refers to a cell's state characterized by excessive production of ROS and is one of the most important regulatory mechanisms for cancer [22].

To protect from high ROS levels, living organisms possess an enzymatic antioxidant that scavenges them. In a normal situation, ROS can be detrimental when produced in high amounts in the intracellular compartments and cells generally respond to ROS by upregulating antioxidants such as superoxide dismutase (SOD), catalase (CAT), glutathione peroxidase (GPx), and glutathione S-transferase (GST) that protect them by converting dangerous free radicals to harmless molecules. Glucose-6-phosphate dehydrogenase (G6PDH) is also involved in the antioxidant mechanism as it produces NADPH which is a direct scavenger of free radicals [23]. ROS and cellular oxidant stress have long been associated with cancer processes [24, 25]. In cancer cells, oxidative stress has been linked to the regulation of numerous cellular processes including DNA damage, proliferation, cellular adhesion, and migration and the regulation of cell survival or death [26].

Previous studies have shown that ROS induce depolarization of the mitochondrial-membrane potential as well as release of cytochrome c from the mitochondria into the cytosol. This increase triggers the activation of caspase-9 and initiates the caspase cascade, which induces apoptosis in tumor cells [6, 7, 27]. Recently, many reports have shown that components from plants such as celastrol [28, 29] and jacaranone [30] induce apoptosis of melanoma cells through production of ROS.

The aim of this study is to observe the effect of MA in the absence of fetal bovine serum (FBS) in B16F10 melanoma cells by analyzing the ROS production and the activity and expression of the main antioxidants enzymes considering that the absence of FBS during growth causes a clear situation of cellular stress.

## 2. Materials and Methods

### 2.1. Drug

**Figure 1:**
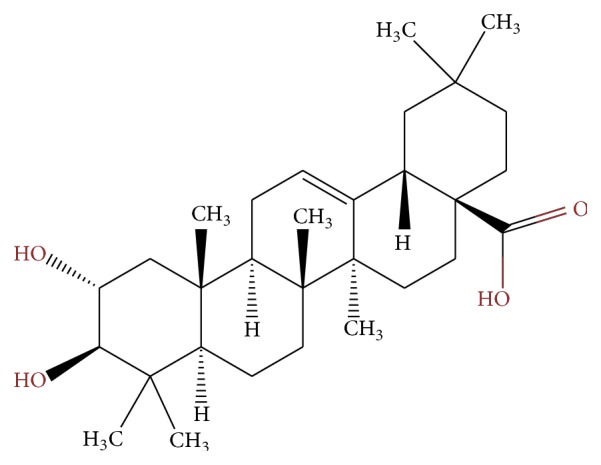
The chemical structure of maslinic acid (MA).

MA was kindly provided by Biomaslinic S.L. Its molecular weight is 472.7 g/mol.  Figure 1 shows the chemical structure of MA ((2*α*,3*β*)-2,3-dihydroxyolean-12-en-28-oic acid). The extract used is a chemically pure white powder comprising 98% MA and is stable when stored at 4°C. MA was dissolved before use at 10 mg/mL in 50% DMSO and 50% PBS. Stock solution was frozen and stored at −20°C. For treatments, this solution was diluted in cell culture medium.

### 2.2. Cell Line and Experimental Conditions

B16F10 cells were cultured in high-glucose DMEM supplemented with 2 mM glutamine, 10% heat-inactivated FBS, 10,000 units/mL penicillin, and 10 mg/mL streptomycin. The cell line was maintained in a humidified atmosphere with 5% CO_2_ at 37°C. Cells were passaged at preconfluent densities by the use of a solution containing 0.05% trypsin and 0.5 mM EDTA. The B16F10 cells were seeded in the culture dishes at the desired density. After 24 h hours, when the cells are attached to the dish, the dishes were incubated with DMEM containing 0% FBS or 10% FBS for 24 h under the conditions described for cell culture. Following that, cells were incubated with MA.

### 2.3. Cell Proliferation

The assay was performed by a variation of the method described by Mosmann [31]. Samples containing 200 *μ*L cell suspension (1.5 · 10^3^ cells/well) were cultured in 96-well plates. Subsequent to the adherence of the cells within 24 h of incubation with and without FBS at 37°C, different MA dilutions on a scale of 10 *μ*g/mL to 100 *μ*g/mL were added separately. Following incubation for 24 h at 37°C in a humidified incubator with 5% CO_2_, MTT dissolved in PBS and medium at 5 mg/mL and sterile-filtered was added to all the wells at a final concentration of 0.5 mg/mL. Following 2 h incubation, the generated formazan was dissolved with 100 *μ*L DMSO per well. The optical density was measured on an ELISA plate reader (ELISA, ELx800, Bio-Tek) at 550 nm. Absorbance was proportional to the number of cells. The concentrations that caused 50% of inhibition of cell growth (IC_50_) were calculated.

### 2.4. Protein Extraction

For sample preparation, cells at 70% confluence were incubated for 24 h without FBS and subsequently were incubated with IC_50_, IC_50/2_, and IC_50/8_ concentrations for 24 h. Following this, the cells were washed three times with PBS, scraped off with a cell scraper (Renner), and collected in 0.5 mL RIPA buffer. Immediately, cells were sonicated on ice for 5 min and maintained by moderate shaking at 4°C for 1 h. Every 15 min, the samples were moderately shaken in a vortex. The lysates were spun in a centrifuge at 10,000 g at 4°C for 15 min. The supernatants were used for enzyme activity and Western blot assays, and the protein concentrations were measured by BCA Protein Assay (Thermo Scientific, USA). For each experimental group, 2 replicates of the homogenates were made. Each replicate was made with 3 different cell populations.

### 2.5. Mitochondrial-Membrane Potential by DHR 123

Changes in the mitochondrial-membrane potential can be examined by monitoring the cell fluorescence after double staining with rhodamine 123 (Rh123) and propidium iodide (PI). Rh123 is a membrane-permeable fluorescent cationic dye that is selectively taken up by mitochondria directly proportional to the MMP (mitochondrial-membrane permeabilization). Around 4 × 10^5^ cells/well were placed on 6-well plates with 2 mL of medium without FBS and treated with cytotoxic compounds for 24 h at IC_50_, IC_50/2_, and IC_50/8_ concentrations. Following the treatment, the medium was removed and a fresh medium with DHR, at a final concentration of 5 *μ*g/mL, was added. After 30 min of incubation, the medium was removed and the cells were washed and resuspended in PBS with 5 *μ*g/mL of PI. The intensity of fluorescence from Rh123 and PI was determined using an ACS flow cytometer (Coulter Corporation, Hialeah, FL, USA), at the excitation and emission wavelengths of 500 nm and 536 nm, respectively.

### 2.6. Enzyme Assays

All enzyme assays were carried out at 25°C using a Power Wave X microplate scanning spectrophotometer (Bio-Tek Instruments, USA) and run in duplicate in 96-well microplates. This made it necessary to adapt all the enzymatic methods described below to the microplate reader to obtain optimal activities. Adaptation of the methods was done by scaling down the reaction mixtures to a final volume of 200 *μ*L and by adjusting both the total time for which reactions were allowed to proceed and the measurement intervals. In addition, the optimal substrate and protein concentrations for the measurement of maximal activity for each enzyme were established by preliminary assays.

The enzymatic reactions were initiated by addition of the tissue extract, except for SOD where xanthine oxidase was used. The millimolar extinction coefficients used for H_2_O_2_, NADH/NADPH, and DTNB [5,5-dithiobis (2-nitrobenzoic acid)] were 0.039, 6.22, and 13.6, respectively. The assay conditions were as follows.

Superoxide dismutase (SOD; EC 1.15.1.1) activity was measured by the ferricytochrome c method using xanthine/xanthine oxidase as the source of superoxide radicals. The reaction mixture consisted of 50 mM potassium phosphate buffer (pH 7.8), 0.1 mM EDTA, 0.1 mM xanthine, 0.013 mM cytochrome c, and 0.024 IU/mL xanthine oxidase. Activity is reported in units of SOD per milligram of protein. One unit of activity was defined as the amount of enzyme necessary to produce a 50% inhibition of the ferricytochrome c reduction rate [32].

Catalase (CAT; EC 1.11.1.6) activity was determined by measuring the decrease of hydrogen peroxide concentration at 240 nm according to Aebi [33]. The reaction mixture contained 50 mm potassium phosphate buffer (pH 7.0) and 10.6 mM of freshly prepared H_2_O_2_.

Glucose-6-phosphate dehydrogenase (G6PDH; EC 1.1.1.49) activity was determined at pH 7.6 in a medium containing 50 mM Hepes buffer, 2 mM MgCl_2_, 0.8 mM NADP^+^ and glucose 6-phosphate were used as substrate. The enzyme activity was determined by measuring the reduction of NADP^+^ at 340 nm as previously described by Lupiañez et al. [34] and Peragón et al. [35]. The change in absorbance at 340 nm was recorded and, after confirmation of no exogenous activity, the reaction started by the addition of substrate.

Glutathione S-transferase (GST; EC 2.5.1.18) activity was measured according to the method described by Habig et al. [36], using 1-chloro-2,4-dinitrobenzene as a substrate.

### 2.7. Western Blot Analysis

Polyacrylamide gel electrophoresis under denaturing conditions (SDS-PAGE) was performed in a Mini-Protean II electrophoresis system (Bio-Rad, Richmond, USA). The cell extract were mixed with a charge buffer that contained 62.5 mM Tris-HCl at pH 6.8, 20 g L^−1^ SDS, 100 mL L^−1^ glycerol, 25 g L^−1^ *β*-mercaptoethanol and 0.045 mM bromophenol blue and then heated for 5 min at 100°C. Polypeptides were separated on a 12% SDS-PAGE and subsequently transferred to polyvinylidene fluoride membranes with a semidry electroblotting system at 1.5 mA/cm^2^ for 45 min in a medium containing 25 mM Tris-HCl, 192 mM glycine, 200 mL L^−1^ methanol, and 1 g L^−1^ SDS. Blots were blocked for 2 h at room temperature with a Tris buffered solution (TBS) that contained 25 mM Tris-HCl, 100 mM NaCl, 2.5 mM KCl, pH 7.6, 1 mL L^−1^ Tween 20, and 15 g L^−1^ bovine serum albumin (BSA) at pH 7.6. Membranes were washed with TBS containing 1 mL L^−1^ Tween 20 (TBS-T) for 15 min and later incubated with specific primary antibodies: anti-glucose 6-phosphate dehydrogenase (G6PDH) 1 : 1,000 (Sigma, A9521), anti-SOD 1 : 500 (Santa Cruz Biotechnology, sc-101523), anti-GST 1 : 1,000 (Santa Cruz Biotechnology, sc-374171), anti-CAT 1 : 1,000 (Santa Cruz Biotechnology, sc-34285), anti-G6PDH 1 : 5,000 (Sigma, A9521), and anti-*α*-actin 1 : 1,000 (Sigma, A2668). Following three washes with TBS-T containing 10 g L^−1^ BSA (TBS-T-BSA) for 10 min, membranes were incubated with a HRP conjugated goat anti-rabbit antibody IgG or anti-mouse IgG (1 : 10,000). Blots were developed using the ECL-Plus Western blot detection system (GE Healthcare). The specific signals were exposed on medical film (Konica Minolta). Films were scanned with a Hewlett-Packard scanner and quantified using Multi-Gauge program (Fuji Film Europe).

### 2.8. Statistical Analysis

Data are shown as mean ± the standard deviation (SD). The statistical significance of differential findings between non-FBS group and control was determined by Student's *t*-test. One-way ANOVA test was performed to determine the significance of the differences between MA concentrations. SPSS version 15.0 for Windows software package was used for statistical analysis. *P* values smaller than 0.05 were considered statistically significant.

## 3. Results and Discussion

### 3.1. FBS Deprivation and Cellular Growth

We examined the effect of MA on the proliferation of B16F10 melanoma cell lines using MTT assay under the presence and absence of FBS. Several groups have studied the effects of the absence of FBS in cancer cells. However, their focus has not been on the variation of cytotoxicity of a compound but on the synchronization of the cells in a particular phase of cell cycle as it was reported that the deprivation of FBS allows arrest of cell cultures into G_0_/G_1_ cell cycle stage [37]. It has been shown that lower concentrations of FBS (0.5% and 0.75%) caused a significant decrease in cellular quantity and this condition could induce DNA fragmentation and subsequent cell death [38]. Moreover, several studies have been focused on improving the composition of FBS in order to enhance the culture cell growth; in this sense, FBS dose dependent studies have been performed [39].

**Figure 2:**
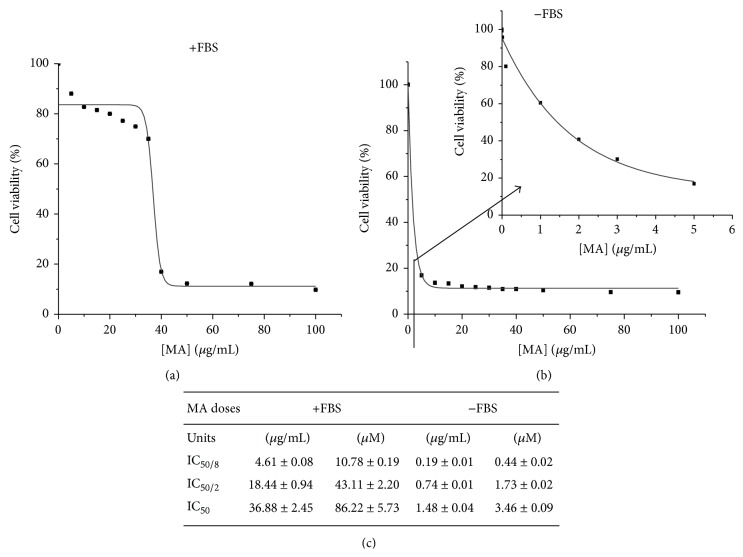
The effect of MA on B16F10 murine melanoma cell viability in the presence of FBS (a) and in the absence of FBS (b). MA cytotoxic doses are shown in (c). Cell proliferation was determined by MTT assay. Values are expressed as means ± SD.

Percentage of living cells (viable formazan accumulating cells) decreased in a dose dependent manner in the presence and absence of FBS. We noticed that 50% growth inhibition values (IC_50_) in response to MA were different in media without FBS, compared to media supplemented with FBS; therefore, MA has an increased cytotoxic effect when cells do not have FBS. These results are reported in Figure 2.

MA dose, which reaches IC_50_ value in the culture medium supplemented with 10% of FBS, was 36.88 *μ*g/mL (86 *μ*M) and 1.48 *μ*g/mL (3.5 *μ*M) in a medium with 0% of FBS. These results indicate that MA has a cytotoxic effect with FBS in the cell culture, but this effect is higher in its absence. Our explanation of this is that absence of FBS modifies the cell homeostasis causing an increase of free radicals and therefore, MA presents a higher cytotoxic effect in the absence of FBS. An imbalance between free radicals and antioxidants results in a condition known as oxidative stress, which leads to metabolic malfunctions and damage to biological macromolecules [40].

MA cytotoxicity has been studied by other groups in several types of cancer cells and it has been shown that the MA quantity has a variable effect on the function of the type of cells and experimental conditions. For example, in HT29 cells the MA quantity that results in growth inhibition of 50% of the population is 30 *μ*M [8, 10] and in Caco-2 the IC_50_ of MA is 10.82 *μ*M [7], whereas in bladder cancer the IC_50_ value changed according to the bladder type cell lines, the values being between 20 and 300 *μ*M [41].

### 3.2. Maslinic Acid Effect on Mitochondrial-Membrane Potential

**Figure 3:**
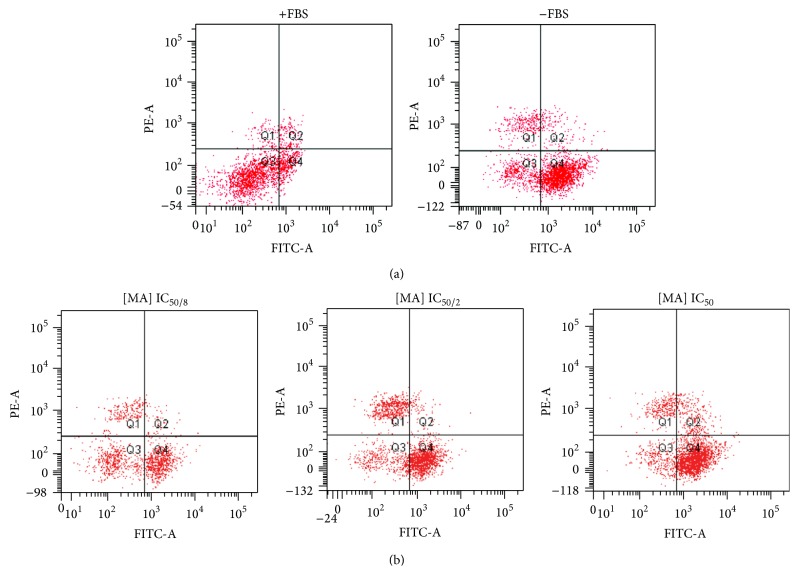
Positive fluorescent Rh123 on B16F10 cells with or without FBS (a) and after MA treatment at different dosages without FBS (b).

DHR 123 is a ROS assay in which this compound can be oxidized intracellularly by ROS to form the positively charged fluorescent Rh123 [42] and is accumulated into the mitochondria [43]. We have performed an assay of ROS production by DHR in the absence of FBS and using three different MA levels (IC_50/8_, IC_50/2_, and IC_50_) based on our cytotoxicity results. Firstly, we have shown that the absence of FBS produced an increase in ROS production due to mitochondrial disruption (Figure 3(a)). FBS composition includes proteins, growth factors, hormones, and ions [44, 45]. Cell culture without FBS forces the cell to synthesize all required compounds necessary for its normal metabolic activity. This fact involves an increase in the cellular metabolism which is reflected in a higher cellular mitochondrial activity, indirectly determined by DHR 123 assay as shown in quadrant 4 (Q4).

Our results indicated that ROS level decreased at lower concentration of MA used (IC_50/8_). Increasing MA induced a direct increase in ROS level; hence, a dose dependent response was observed (Figure 3(b)). Among other properties, MA has been reported as a natural antioxidant [46–49]. In this context, the decrease in ROS level observed at MA at IC_50/8_ concentration could be attributed to its antioxidant property. On the other hand, MA induces apoptosis by activating intrinsic pathways that increase cellular ROS production [4, 48–52]. Mitochondria are the primary cellular site of ROS production and, under certain conditions, elevated mitochondrial ROS levels can serve as proapoptotic signals in cancer cells; as a result, drugs that induce ROS are receiving greater attention for their potential as chemotherapeutic agents [53]. In our previous studies, we have shown that MA induces apoptosis in cancer colon cells [10, 13, 54].

In the present study, the observed results on ROS production at higher MA levels are not just probably due to the absence of the antioxidant effect but due to a combination of antioxidant and apoptotic properties of MA when cells are cultured without FBS. Therefore, the increased MA levels are related to the higher apoptotic capacity of triterpene as shown by the higher ROS levels determined by DHR 123 assay (Figure 3).

### 3.3. Antioxidant Enzymatic Response

**Figure 4:**
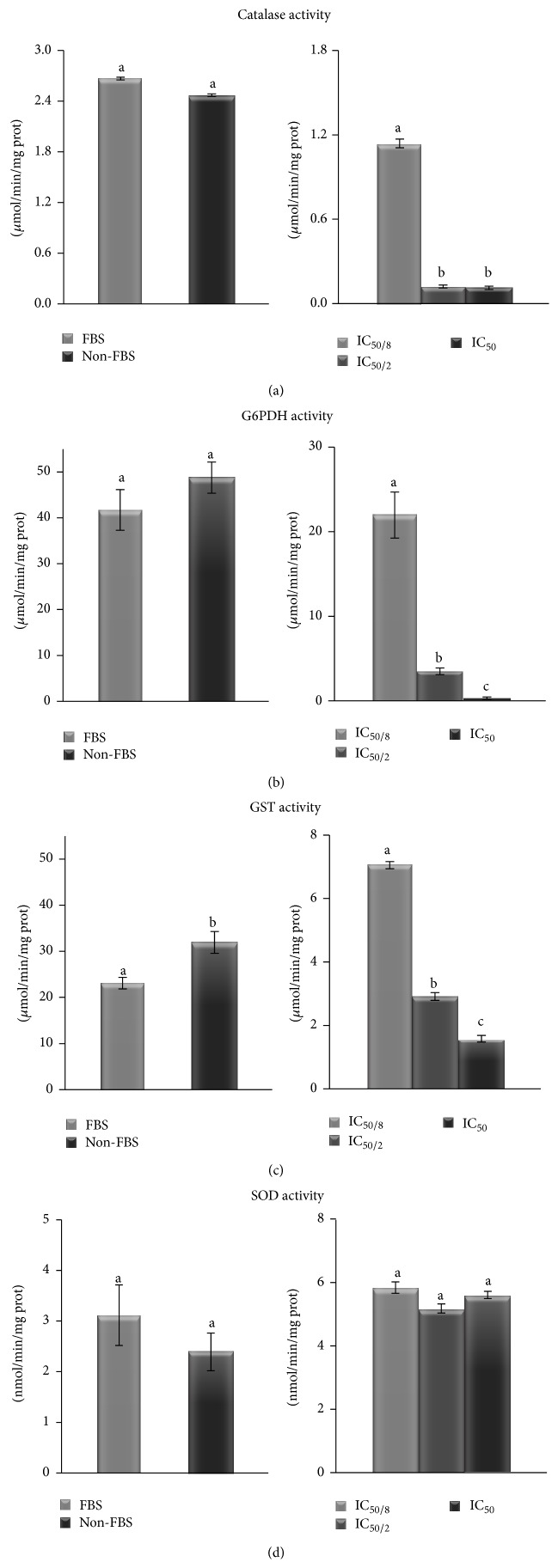
CAT (a), G6PDH (b), GST (c), and SOD (d) specific activities on B16F10 cells. On the left side, cells cultivated with and without FBS, and on the right side, cell cultivated without FBS and different MA dosages. Values are expressed as means ± SD. Different letters indicate significant differences (*P* < 0.05).

An evaluation of the activities of the antioxidant enzymes superoxide dismutase (SOD), catalase (CAT), glucose 6-phosphate dehydrogenase (G6PDH), and glutathione-S-transferase (GST) was performed under our experimental conditions (Figure 4). The results indicated that CAT, G6PDH, and SOD showed no change in their activity in the absence of FBS compared to the control with FBS, and only GST activity increased in the absence of FBS with respect to control in the presence of FBS, since this enzyme is related to detoxification of lipid peroxidation products, which are being generated under the assayed conditions.

When MA is added to the cell culture, CAT, G6PDH, and GST activities decreased below controls data. This effect was observed at all doses of MA used and in a dose dependent response pattern. However, SOD activity in the presence of MA was not affected. This variability in responses of different antioxidant enzymes may be due to linked factor combination. As previously indicated, MA induces apoptosis at higher doses. Apoptosis mechanism involves biochemical changes in the cells to achieve death. The changes observed in the first step of apoptosis imply homeostatic cellular disruption.

These alterations inhibit biosynthesis of new molecules and damaged metabolites renewal, a fact related to the low G6PDH activity observed. G6PDH is the main enzyme to produce NADPH which is essential for reductive biosynthesis and nucleic acid synthesis [55] and protects the cell against oxidants [56]. Among other biological properties of NADPH, it has been demonstrated that it protects CAT from inactivation [57–59] and plays a crucial role in maintaining the redox state of the cell through GSH regeneration from GSSG. Low NADPH levels due to the imbalance in the cellular homeostasis could be, partially, responsible for the low activity levels observed in CAT and GST enzyme activities. All these results are in accordance with DHR 123 assay.

### 3.4. Validation by Western Blot

**Figure 5:**
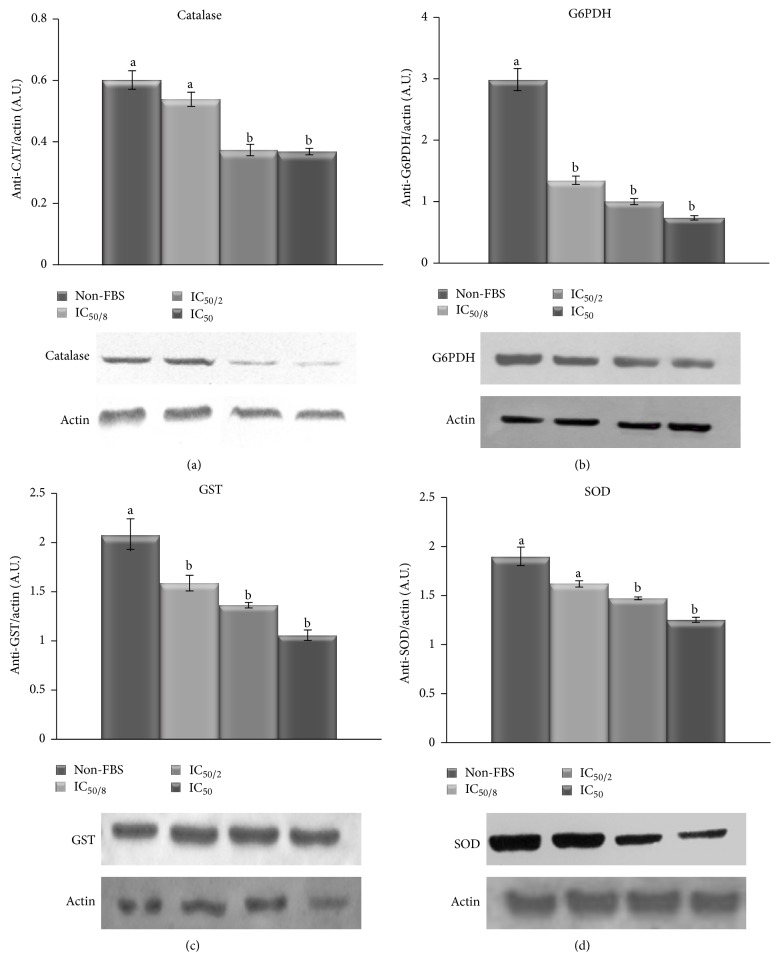
Western blots of CAT (a), G6PDH (b), GST (c), and SOD (d) on B16F10 cells cultivated without FBS at different MA dosages. The levels of specific protein expression are shown as arbitrary intensity units of each band compared to arbitrary intensity units of actin. Values are expressed as means ± SD. Different letters indicate significant differences (*P* < 0.05).

To corroborate the effect of different MA levels in cells without FBS in the antioxidant enzymes measured, an immune-blotting analysis was carried out to confirm the differential expression of same specific enzymes: CAT, G6PDH, GST, and SOD (Figure 5), under the same experimental condition (MA levels without FBS). Results obtained of immune-blotting analysis agree with those in the enzyme analysis, tending to decrease their activity and expression as MA concentration increases without FBS, although this pattern is not statistically significant in all enzymes.

## 4. Conclusions

It has been described in the scientific literature that triterpenes and polyphenols, in physiological doses, are able to improve the intrinsic cell tolerance against oxidative stress by increasing the antioxidant potential and modulation related to proliferation/survival in cell cultures and experimentation animals. However, at supraphysiological doses, these compounds appear to induce programmed death via the activation of signals involved in apoptosis and inhibition of proliferation proteins associated with survival/cell death. This indicates that the experimental conditions (concentrations, culture conditions, cell type, duration of the treatment, etc.) have to be seriously considered as they may determine the biological activity of these natural compounds. As it is difficult to predict their effect, there is a need to understand their molecular mechanisms of action in each context. Results obtained in this paper must be considered in these experimental conditions.
